# Anesthetic Considerations and Outcomes in Amniotic Fluid Embolism: A Retrospective Study over a 15-Year Period

**DOI:** 10.3390/jcm13102916

**Published:** 2024-05-15

**Authors:** Carolina Susanu, Anamaria Harabor, Petronela Vicoveanu, Ingrid-Andrada Vasilache, Alina-Mihaela Călin

**Affiliations:** 1 Clinical and Surgical Department, Faculty of Medicine and Pharmacy, ‘Dunarea de Jos’ University, 800216 Galati, Romania; cc574@student.ugal.ro (C.S.);; 2 Department of Mother and Newborn Care, Faculty of Medicine and Biological Sciences, ‘Ștefan cel Mare’ University, 720229 Suceava, Romania; 3 Department of Mother and Child Care, “Grigore T. Popa” University of Medicine and Pharmacy Iasi, 700115 Iasi, Romania; ingrid-andrada.vasilache@umfiasi.ro

**Keywords:** embolism, amniotic fluid, cardio-pulmonary resuscitation pregnancy, critical care

## Abstract

**(1) Background:** A rare and unexpected consequence of childbirth, labor, or the immediate postpartum period is amniotic fluid embolism (AFE). This study aims to identify AFE cases during or immediately after birth from anesthetic management perspectives. Secondary goals include assessing patient clinical features, obstetric care techniques, birth outcomes, and case survival. **(2) Methods:** A retrospective observational study assessed AFE patients hospitalized in three Romanian clinical institutions from October 2007 to April 2023. Based on the Society of Maternal-Fetal Medicine (SMFM) criteria, we diagnosed 11 AFE patients. **(3) Results:** AFE occurred in eight cases (73%) during peripartum, two (18%) within 30 min after placental delivery, and 1 (9%) during a scheduled cesarean surgery. Only one of six cardiorespiratory arrest patients responded to external cardiac massage, while the other five (83%) needed defibrillation. The patients received, on average, five units of red blood cells, six of fresh frozen plasma, and two of activated platelets. Six patients (55%) received factor VIIa infusions. Maternal mortality was 36.3%. Six neonates (75%) needed neonatal resuscitation, and two (25%) died on the second and third days. **(4) Conclusions:** AFE management necessitates a multidisciplinary approach and the incorporation of advanced life support techniques to optimize outcomes for both the mother and newborn.

## 1. Introduction

Amniotic fluid embolism (AFE) represents an exceptionally uncommon complication in obstetrics, typically manifesting during labor, delivery, or shortly after childbirth [1]. Due to its infrequent occurrence and the absence of a global agreement on diagnostic standards, the underlying mechanisms of this condition remain inadequately elucidated [2]. Reported occurrences of AFE in developed nations vary between 1.9 and 6.1 cases per 100,000 births, yet the diagnostic criteria exhibit significant heterogeneity, possibly leading to an overestimation of its incidence [3].

The exact mechanisms underlying AFE remain elusive. It is hypothesized that the infiltration of amniotic fluid, which carries fetal cells and antigenic substances, into the maternal bloodstream via a disruption in the maternal-fetal interface triggers abnormal activation of humoral and immunological pathways. This process results in the release of vasoactive and procoagulant agents, resembling systemic inflammatory response syndrome (SIRS) [4]. Additionally, bacterial antigens may vie with fetal antigens, contributing to the development of AFE.

Consequently, there is an abrupt rise in pulmonary and right ventricular (RV) pressures, leading to the functional impairment of the right ventricle and, subsequently, of the left ventricle, culminating in circulatory collapse. Left ventricular (LV) failure may also stem directly from hypoxic injury, the release of maternal inflammatory agents, or the depressant impact of amniotic fluid [5].

Acute pulmonary hypertension results in a significant disruption of the ventilation/perfusion ratio, marked by the onset of cardiogenic pulmonary edema and hypoxemic respiratory failure [6]. Subsequently, non-cardiogenic pulmonary edema may develop in certain individuals [7]. Examination of lung tissue during autopsy, utilizing cytokeratin staining, typically unveils pulmonary mast cells and stromal cells, along with sporadic presence of fetal cells surrounding pulmonary capillaries and inflammatory cells within the alveoli.

Activation of Factor VII and platelets, along with the release of inflammatory mediators, triggers the coagulation cascade, leading to disseminated intravascular coagulation (DIC), ischemia, and multi-organ failure [8]. Hemorrhaging from DIC exacerbates hemodynamic instability.

In 2016, the Society of Maternal-Fetal Medicine (SMFM) endeavored to offer evidence-based guidelines for diagnosing and managing suspected AFE [4]. These guidelines advocated resuscitation following advanced life support (ALS) protocols for patients experiencing cardiac arrest, engaging a multidisciplinary team, ensuring appropriate ventilation and circulatory support, addressing coagulopathy induced by AFE, and promptly delivering fetuses beyond the point of viability.

The SMFM diagnostic criteria for AFE comprise the following mandatory components [4]:

- The abrupt occurrence of cardiorespiratory arrest OR arterial hypotension (systolic blood pressure < 90 mmHg) accompanied by signs of respiratory dysfunction (such as dyspnea, cyanosis, or peripheral oxygen saturation < 90%).
- Recording disseminated intravascular coagulation (DIC) according to the criteria established by the Scientific and Standardization Committee for DIC of the International Society of Thrombosis and Haemostasis (ISTH) [9], adjusted for pregnancy:
  ○ Platelet count (>100,000/mL = 0 points; <100,000 = 1 point; <50,000 = 2 points).
  ○ Extended prothrombin time or international normalized ratio (<25% increase = 0 points; 25 to 50% increase = 1 point; >50% increase = 2 points).
  ○ Fibrinogen level (>200 mg/dL = 0 points; <200 mg/dL = 1 point. A score of ≥3 indicates compatibility with the diagnosis of DIC.
- Clinical onset either during labor or within 30 min of placental expulsion.
- Absence of fever (≥38 °C) during labor.

In the majority of patients (90%), the emergence of distinctive clinical signs associated with AFE is sudden and swiftly deteriorating [10]. Typically, they exhibit sudden hypoxia and arterial hypotension, frequently followed by noncardiogenic pulmonary edema and hemorrhage due to DIC. For patients encountering AFE during cesarean delivery, the onset of these symptoms can be postponed with anesthetic interventions targeting the early correction of vital sign alterations. Clinical presentations encompass the following [10]:

- Aura—Up to one-third of individuals might encounter abrupt feelings of anxiety, chills, nausea and vomiting, agitation, or alterations in mental state just prior to the event.
- Cardiorespiratory failure and/or arrest—Most patients experience a sudden onset of hypoxemic respiratory failure, hypotension leading to cardiogenic shock, and/or cardiovascular collapse or cardiac arrest. Common clinical indicators comprise reduced oxygen saturation, dyspnea, tachypnea, cyanosis and sometimes wheezing. Cardiac arrest typically arises from sustained pulseless ventricular tachycardia or ventricular fibrillation, although it can also stem from bradyarrhythmia and/or asystole. Should the patient survive the initial cardiorespiratory episode, they frequently develop non-cardiogenic pulmonary edema as the left ventricular failure ameliorates.
- Hemorrhaging—In DIC, bleeding occurs in over 80% of AFE patients, typically manifesting shortly after the onset of cardiorespiratory compromise. In postpartum confinement, extended bleeding from puncture or intervention sites may indicate the onset of DIC.
- Tonic–clonic seizures and/or stroke—These initial presentations are uncommon and less frequently encountered complications of AFE.

Managing AFE during pregnancy poses challenges due to the unique hemodynamic characteristics inherent to gestation, the varied clinical presentation, and the presence of diverse risk factors. Additionally, the impact of individual risk factors on the progression of AFE remains incompletely understood. The majority of maternal deaths after an amniotic fluid embolism occur in the first hours after diagnosis, and clinicians need to have a high degree of suspicion in the presence of suggestive symptomatology, as well as a prompt reaction to clinical signs of cardiopulmonary arrest or severe fetal distress.

The long-term complications, such as neurological sequels, acute kidney failure, cardiac ischemia, recurrent arrythmias, or non-cardiogenic pulmonary edema, need surveillance and individualized management. Also, the neurological impairment of newborns due to prolonged in utero hypoxia needs to be addressed as soon as possible, and these patients should be included in the long-term follow-up by specialized personnel.

Given the rarity of this condition, there is a growing necessity to gather pertinent clinical and paraclinical information to aid clinicians in navigating the intricate management of such cases.

This is the first study in Romania that aimed to underscore the anesthetic approach to AFE cases across three hospital units in the Moldova region. Since medical data about this topic are extremely scarce in our country, we aimed to describe the clinical management and outcomes of AFE cases who presented at tertiary clinics and who benefited from multidisciplinary management.

The primary goal of this study is to depict instances of AFE happening at birth or shortly after delivery, with a particular focus on anesthetic management. Secondary objectives include examining the patients’ clinical characteristics, obstetric management, birth outcomes, and the subsequent survival outcomes of the cases.

## 2. Patients and Methods

We conducted a retrospective observational study involving pregnant or postpartum confinement patients diagnosed with amniotic fluid embolism admitted to three clinical hospitals in Romania: “Buna Vestire” Obstetrics and Gynecology Hospital in Galati, “Cuza-Vodă” Clinical Hospital of Obstetrics-Gynecology in Iași, and “St. Ioan” Emergency Hospital in Suceava, spanning from October 2007 to April 2023.

Ethical approval for this study was received from the Institutional Ethics Committee of the University of Medicine and Pharmacy, ‘Dunărea de Jos’ Galati, Romania (No. 51687/7 December 2022), “Cuza-voda” Clinical Hospital of Obstetrics and Gynecology, Iasi (No. 151/13 February 2022), and from “St. Ioan” Emergency Hospital, Suceava (No. 7/31 January 2022). Informed consent for the retrospective use of personal data was obtained from all participants involved. All research methodologies adhered to international guidelines and regulations.

The criteria for inclusion comprised the following: confirmed diagnosis of AFE, AFE that occurred during birth or shortly after delivery (in the first 30 min after placental delivery), singleton pregnancies, an age of 18 years or above, and comprehensive medical records. The patients’ medical records underwent systematic assessment, and essential clinical data were collected.

The exclusion criteria comprised the following: multiple pregnancies, ectopic pregnancies, abortions during the first and second trimesters, fetal demise in utero, a fetus exhibiting chromosomal or structural abnormalities, intrauterine infection, and incomplete medical records.

Cases of AFE were identified using SMFM criteria. The histopathological examination was complementary. Traditionally, the presence of fetal squamous cells in the pulmonary circulation was used as an instrument for AFE diagnosis. However, this test is now considered suggestive rather than diagnostic, as these cells can also be found in patients without the typical symptomatology of AFE. This can be particularly relevant if, on histopathological examination, the squamous cells are abundant, protected by neutrophils, or accompanied by other fecal debris [11].

The following variables were documented from the medical records of our patients: demographic information, the patient’s medical background, clinical manifestations, laboratory values upon admission, imaging results, pregnancy outcomes, the duration of hospitalization, the requirement for invasive mechanical breathing support, treatment administered, and a survival post-AFE event.

Descriptive analysis was used to present our data, which were expressed as means and standard deviations (SDs) or medians and interquartile ranges for continuous variables and numbers and percentages for categorical data.

We also provided a Cox regression model with the Breslow method for ties in order to identify the clinical and paraclinical predictors for maternal death, and to quantify their impact as a hazard ratio (HR) and 95%CI.

The clinical predictors included in the model were represented by artificial rupture of membranes and complicated delivery (i.e., birth trauma, labor dystocia, and operative delivery).

The paraclinical characteristics included in the model were represented by a prolonged prothrombin time (prothrombin time that exceeds 13 s), severe thrombocytopenia (platelet count less than 50.000/mm^3^), low serum fibrinogen levels (less than 100 mg/dL), severe desaturation (oxygen saturation < 85%), and recurrent arrythmias (i.e., atrial or ventricular tachycardia, bradyarrhythmia, etc).

We have also performed Kaplan–Meier analysis, and the survival estimates were plotted for a chosen time frame of 72 h after giving birth. The median time frame and standard deviation until maternal death were reported.

A *p*-value less than 0.05 was considered statistically significant. These analyses were performed using STATA SE (version 17, 2023, StataCorp LLC, College Station, TX, USA).

## 3. Results

Out of the 13 records identified using our research methodology, 11 satisfied the inclusion criteria aforementioned. Two cases were omitted from the analysis due to inadequate clinical data, preventing us from establishing a strong suspicion of amniotic fluid embolism.

The median maternal age was 24.3 years [range: 20–31], median parity was 1.13 [range: 0–2], and median gestational age was 38 weeks [range: 36 + 2–39 + 5] (Table 1). Among the maternal and fetal comorbidities identified in the reviewed cases, notable conditions included preeclampsia (two cases), intrauterine growth restriction accompanied by preeclampsia (one case), and a medical history of pulmonary microemboli and antiphospholipid syndrome (one case).

In terms of the obstetric context, amniotic fluid embolism occurred in eight cases (73%) during the peripartum period, two cases (18%) within the first 2 h postpartum and one case (9%) during a scheduled cesarean section. Six patients (55%) underwent labor induction using oxytocin (5/6) and/or misoprostol (1/6). In all cases, fetal membrane rupture had occurred spontaneously (45%) or artificially (55% at the time of AFE occurrence). Uterine hypertonia was observed in four patients (40%).

Maternal clinical manifestations indicative of AFE included respiratory symptoms (such as dyspnea oxygen desaturation), cardiovascular symptoms (like syncope, collapse, cardiorespiratory arrest), and/or hemorrhagic symptoms. In each instance, the clinical presentation transitioned to that of prolonged postpartum hemorrhage, with deterioration observed in 91% of cases due to DIC.

Fetal heart rhythm irregularities consistently coincided with maternal deterioration. Obstetric intervention was extended to eight fetuses (those in utero during the critical period). Delivery was successfully accomplished in all instances within 20 min, and in five cases (62.5%) within 5 min of symptom onset, involving seven emergency cesarean sections and one vacuum-assisted extraction.

Postpartum hemorrhage occurred in all patients, necessitating surgical intervention to control bleeding (hemostatic hysterectomy) in seven cases (64%) where medical/surgical hemostasis approaches (high-dose oxytocin, misoprostol, B-lynch suture) were ineffective. Uterine and hypogastric artery ligation was performed in two cases.

All patients necessitated an advanced cardiorespiratory resuscitation protocol. Among the six patients experiencing cardiorespiratory arrest, external cardiac massage proved effective in only one case, with the remaining five (83%) requiring defibrillation.

Blood and blood product transfusions were necessary for all cases, averaging five units of red blood cells, six units of fresh frozen plasma, and two units of activated platelets per patient.

Factor VIIa (Novoseven) infusions were administered to six patients (55%). Among the 11 patients diagnosed with AFE, 4 fatalities occurred (36.3%) due to multiple-organ failure.

**Figure 1:**
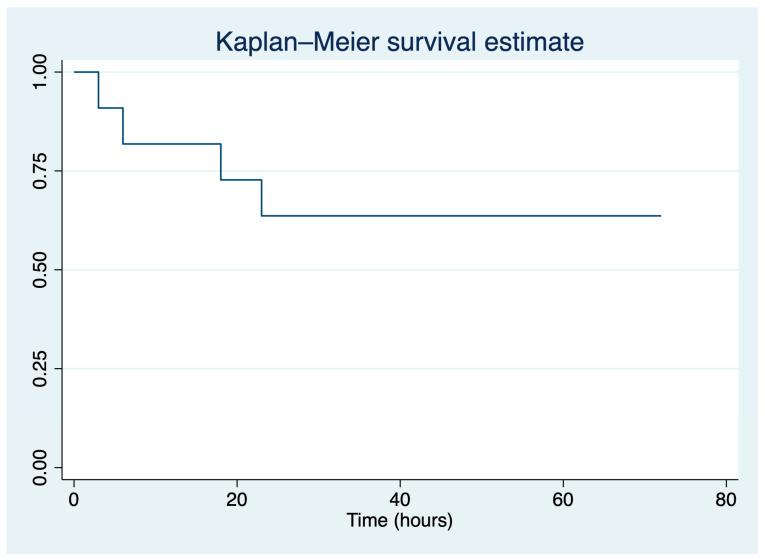
Kaplan–Meier survival estimate for our cohort of patients.

The mean time until death and standard deviation were 12.5 ± 9.53 h, and Figure 1 reflects the Kaplan–Meier survival estimates in a 72 h postpartum period.

The patients who survived the AFE had neurological damage (4 cases, 57.14%), acute kidney injury (1 patient, 14.28%), persistent arrythmias (1 patient, 14.28%), and noncardiogenic pulmonary edema (1 patient, 14.28%).

We performed a Cox regression model with the Breslow method for ties in order to identify clinical and paraclinical predictors of maternal death, and the results are presented in Table 2.

Out of the evaluated predictors, only the artificial rupture of membranes (HR: 2.73; 95%CI: 0.46–7.38, *p* = 0.004), a complicated delivery (HR: 2.32; 95%CI: 0.34–8.61; *p* = 0.001), a prolonged prothrombin time (HR: 1.47; 95%CI: −0.33–5.49; *p* = 0.03), and severe thrombocytopenia (HR: 1.12; 95%CI: −0.77–4.98, *p* = 0.04) significantly increased the risk of maternal death.

Out of the 11 newborns, 5 were male and 6 were female, resulting in a gender ratio of 0.83 (Table 3). The median birth weight was 3120 g, ranging from 2340 g to 3600 g. Six neonates (75%) born to mothers who experienced peripartum AFE required neonatal resuscitation, and two (25%) passed away on the second and third day, respectively.

The median Apgar score was 5 at 1 min and 7 at 5 min. Four neonates scored less than 7 on the Apgar scale at 5 min. The median umbilical arterial pH was 7.15, with 3 neonates having a pH below 7.00 (27%).

In two cases, suspicion of pulmonary thromboembolism was considered as a potential alternative diagnosis, but this was dismissed upon performing computed tomography (CT) pulmonary angiography.

## 4. Discussion

The findings of this retrospective observational study conducted across multiple centers provide valuable insights into the anesthetic management of these cases. Maternal mortality in this study stood at 36.3%, which, while lower than the rates reported in earlier studies (61% between 1988 and 1993) [12], was higher compared with those in more recent studies from developed countries in America or Europe. For instance, Roberts et al. reported a mortality rate of 35% [13], Abenhaim et al. reported 22% [14], and Kramer et al. reported 13% [15].

Our Kaplan–Meier analysis outlined that the mean time until maternal death was 12.5 h. Moreover, the AFE patients who survived had neurological damage, acute kidney injury, persistent arrythmias or noncardiogenic pulmonary edema. Previously, it was recorded that 50% of patients experience mortality during the initial hour, and approximately two-thirds experience mortality within 5 h following the incident, with a significant prevalence of severe and lasting neurological impairment among those who survive [16].

The variations in these mortality rates might be elucidated by the diverse approaches to anesthetic management, with certain advanced centers benefitting from immediate multidisciplinary case oversight and access to critical technologies such as ECMO (extracorporeal membrane oxygenation) for maintaining vital functions. Conversely, the timely suspicion of AFE based on the SMFM criteria and the prompt admission of patients to intensive care units have contributed to a decline in AFE mortality compared with historical rates, which were over 50% higher a few decades ago. Additionally, Dennis et al. underscored the imperative of multidisciplinary collaboration and the application of advanced life support techniques to mitigate maternal mortality attributed to AFE [17].

The neurological sequels are the most prevalent amniotic fluid complications reported in observational studies, affecting between 24% and 50% of cases [6,18,19]. The neurological sequels are closely followed by renal failure [20,21], cardiovascular and pulmonary disorders [22,23], or infectious complications [24].

Consumption coagulopathy was consistently observed in association with AFE, posing challenges in its management, which was guided by national protocols. In seven cases, conservative measures to address uterine atony proved ineffective, necessitating hemostatic hysterectomy with hypogastric artery ligation.

The majority of the literature data regarding the association between DIC and AFE is provided by case reports, and only a handful of retrospective studies outlined this associations. One of these studies was conducted by Ponzio-Klijanienko et al., and it retrospectively assessed the clinical presentation of 14 women with a strong suspicion of AFE identified during a 12-year time frame, as well as the validity of SMFM criteria in this group of patients [25]. Data from this study indicated that clinical DIC occurred in all evaluated patients, with a median platelet count of 65,550/mm^3^, a median pro-thrombin time of 34%, and a median serum fibrinogen of 0.4 g/L.

Moreover, Gilbert et al. conducted a population-based study in a cohort of 1,094,248 deliveries during a 2-year period, and identified only 53 cases of AFE, outlining a population frequency for this disorder of 1 per 20,646 pregnancies [26]. Their reported rate of DIC secondary to AFE was 66% (35 patients), and the rate of DIC encountered in patients who later died was 79% (11/14 patients).

Other pathology, characterized by sudden hypoxia/hypotension then followed by a coagulopathy in pregnancy, is represented by peripartum hemorrhagic shock, which can occur as a consequence of uterine atonia, placenta praevia, uterine rupture or abruptio placentae [27]. Other differential diagnoses for AFE that can manifest with hypoxia, hypotension and/or consumption coagulopathy include thrombotic or air pulmonary embolism, acute myocardial infarction or heart failure, anesthetic complications or anaphylactic shock, and eclampsia [27]. All these differential diagnoses should be carefully analyzed and excluded, especially considering that the AFE diagnosis is clinical, as stated by the SMFM guideline [4].

Yoneyama et al., in a retrospective case study analysis conducted in a 29-year period in three hospitals from Japan, investigated the clinical characteristics and risk factors for AFE [28]. In the cited study, the authors used the Clark criteria [6,29] for the AFE diagnosis and identified 10 cases with this pathology, outlining an incidence of 4.8 per 100,000 live births. The DIC was encountered in all these patients, and only three patients (30%) survived, with two of them experiencing a form of neurological damage, while one patient experienced acute kidney failure.

An alternative conservative treatment option, uterine artery embolization through interventional radiology techniques, was not accessible in any of the examined facilities. This method has demonstrated efficacy in numerous clinical trials for managing uterine atony across various scenarios. Its integration into clinical practice could broaden the range of therapeutic options available at the institutional level [30,31,32].

The complete understanding of clinical risk factors for AFE remains incomplete in the literature. In the cases reviewed in this study, we identified pre-eclampsia in two cases, intrauterine growth restriction associated with preeclampsia in one case, and thrombotic events and adverse obstetric histories in one case.

Every patient experienced ruptured membranes, either spontaneously or artificially, at the time of AFE occurrence, which pose an additional risk factor. Previous research has indicated that damage to the maternal vascular bed within the context of placental ischemic disease could serve as a predisposing factor for AFE initiation [3,22,33].

The distress experienced by in utero fetuses during an AFE has potential negative consequences on further adaptation to extrauterine life. Among our cohort of newborns, two fatalities occurred (18.18%), resulting in a mortality rate consistent with findings from other studies [34,35]. To enhance neonatal outcomes, it is imperative to promptly manage cases and maintain a heightened level of suspicion for AFE in situations of sudden maternal decompensation during the peripartum or postpartum period. However, it is important to keep in mind that AFE appears suddenly, and clinicians have very little time to complete the delivery so that the fetal outcomes are favorable. This aspect points out the need to a strong collaboration between the members of labor ward and obstetrical personnel.

Finally, our study highlighted the clinical and paraclinical predictors for maternal death, and showed a significant impact of the artificial rupture of membranes, a complicated delivery, a prolonged prothrombin time, and severe thrombocytopenia. Our results are in line with previously published data regarding predictors of maternal death in the context of AFE.

Thus, Han et al. conducted an observational retrospective study and included patients with or without amniotic fluid embolism that gave birth in the Second Affiliated Hospital of Kunming Medical University in a 15-year period [36]. Their results indicated that artificial rupture of membranes, also known as amniotomy, the use of f oxytocin and misoprostol for labor induction, precipitate delivery, obstetrical trauma and increased levels of IL-6 and IL-8 determined in the amniotic fluid were significant predictors for maternal death in AFE patients. Prolonged prothrombin time, severe thrombocytopenia, and low fibrinogen levels were also confirmed as predictors for maternal death in the context of AFI [37,38,39].

The study’s limitations include the small patient cohort and its retrospective nature. However, the study is notable for showcasing the clinical expertise from three medical institutions in managing an exceedingly rare condition, AFE, which demands a multidisciplinary approach. Therefore, longitudinal studies involving larger patient populations will be necessary to validate the associations between different clinical risk factors and the incidence of AFE.

Current research evaluated the feasibility of various diagnostic markers for AFE. The most studied markers include elevated zinc coproporphyrin, a component of meconium, and serum sialyl-Tn, a fetal antigen present in both meconium and amniotic fluid [40,41]. Other biomarkers include decreased levels of C3, C3a, and C4, insulin-like growth factor binding protein, interleukins (IL) −6 and −8, tumor necrosis factor (TNF) alpha, endothelin, activin A and proopiomelanocortin (POMC) [42,43]. A prospective design for further studies on this pathology is difficult given its rarity and complex clinical presentation, but retrospective analyses could be performed in blood serums and/or histopathological probes stored in various biobases, and they could include novel biomarkers in order to evaluate their diagnostic accuracy and feasibility for use in clinical practice.

This study has the particularity of presenting AFE cases who benefited from a multidisciplinary approach in an almost 16-year period, and highlights the importance of prompt intensive care support that limits adverse pregnancy and neonatal outcomes. The analyzed data were collected from two tertiary maternity centers specializing in complex pathologies and utilizing a prompt multidisciplinary approach. On the other hand, the health care coverage for obstetrical patients who had an episode of AFE in isolated regions of Romania is poor, and its true incidence can be underestimated. Thus, clinicians should have a high degree of suspicion when encountering pregnant patients with symptomatology suggestive of AFE, and should immediately ask for a multidisciplinary team for further management.

Nonetheless, from the basic life support of airways, breathing and circulation to more advanced techniques or respiratory and circulatory support using orotracheal intubation and vasopressors, it is important to recognize AFE timely and to work in a multidisciplinary team for improving obstetrical and neonatal outcomes, as well as good clinical practices [44].

The incidence rates of AFE are variably reported in the literature, depending on the source data (national registries, single-center versus multicentrer studies, addressability to a specific center, etc.). Stein et al. gathered data from the National Hospital Discharge Survey from United States for determining the incidence of AFE, and indicated an incidence of 22 cases of AFE for 100,000 cesarean deliveries, as well as a general incidence of 11 AFE cases per 100,000 patients [45]. The majority of the patients included in this study have undergone cesarean delivery, and this surgical procedure is cited in the literature as a risk factor for the occurrence of AFE [46,47].

Long-term follow-up of both surviving mothers and newborns is essential for the further diagnosis of neurological, renal, cardiac and pulmonary events. Moreover, further studies could outline the clinical and paraclinical predictors for these adverse outcomes and could provide risk stratifications systems that will allow clinicians to identify those patients at risk for other health problems.

## 5. Conclusions

Amniotic fluid embolism is an exceptionally rare condition that carries significant implications for maternal, fetal, and neonatal health outcomes.

The management of AFE necessitates a multidisciplinary approach and the incorporation of advanced life support techniques to optimize outcomes for both the mother and newborn.

Despite advancements in anesthesia and intensive care, the maternal mortality rate attributable to AFE remains elevated. It is imperative to underscore the individual risk profiles of patients, aiding clinicians in anticipating these complications and providing optimal therapeutic interventions.

## Figures and Tables

**Table 1:** IUGR—intrauterine growth restriction. APS—antiphospholipid syndrome.

Pregnant Women Data		Median and IQR Range/Mean and SD/Number of Patients and %
Demographics	Maternal age, years	24 (20–31)/24.4 ± 6.2
	Living Environment	Rural = 7 (63.6%) Urban = 4 (36.6%)
	Age of AFE diagnosis during pregnancy, weeks	38 (36 + 2–39 + 5)/38.1 ± 1.3
Clinical parameters	Preeclampsia	Yes = 2 (18.1%)
	IUGR	Yes = 1 (9%)
	APS	Yes = 1 (9%)
	Thrombotic antecedents	Yes = 1 (9%)
	Smoking	Yes = 2 (18.1%)
	Parity	1 (1–2)/1.13 ± 0.88

**Table 2:** Results from the Cox regression model with the Breslow method for ties.

Predictor	HR and 95%CI	*p* Value
Artificial rupture of membranes	2.73 (0.46–7.38)	0.004
Complicated delivery	2. 32 (0.34–8.61)	0.001
Prolonged prothrombin time	1.47 (−0.33–5.49)	0.03
Severe thrombocytopenia	1.12 (−0.77–4.98)	0.04
Low fibrinogen	0.99 (0.31–2.47)	0.45
Severe desaturation	0.82 (0.42–1.60)	0.57
Recurrent arrythmias	1.04 (−0.67–2.88)	0.78

Legend: HR—hazard ratio; CI—confidence interval.

**Table 3:** Neonatal outcomes in the evaluated cohort.

Neonatal Outcomes	Patients Admitted to the NICU or Clinical Ward (n = 11 Patients)
Sex (n/%)	Male = 5 (45.4%) Female = 6 (54.5%)
Birth weight (g)	3120 (2340–3600)
Apgar score at 5 min	7 (5–8)
Hospitalization to NICU	Yes = 2 (18.1%)
Arterial pH	7.15 (6.71–7.32)
Neonatal death	Yes = 2 (18.1%)

Legend: NICU—neonatal intensive care unit.

## Data Availability

The datasets produced and/or analyzed in the present study can be obtained from the corresponding author upon a request made in a reasonable manner.
